# Bleeding and Thromboembolic Events in Patients with Non-Valvular Atrial Fibrillation Treated with Apixaban or Rivaroxaban

**DOI:** 10.21315/mjms2022.29.2.15

**Published:** 2022-04-21

**Authors:** Ahmad Salihin ABDULLAH, Hwee Pheng TAN, Shamin MOHD SAFFIAN

**Affiliations:** 1 Centre for Quality Management of Medicines, Faculty of Pharmacy, Universiti Kebangsaan Malaysia, Kuala Lumpur, Malaysia; 2 Pharmacy Department, Universiti Kebangsaan Malaysia Medical Centre, Kuala Lumpur, Malaysia

**Keywords:** Factor Xa inhibitors, atrial fibrillation, thromboembolism, haemorrhage

## Abstract

Rivaroxaban and apixaban are effective oral anticoagulants, but their usage has been associated with an increased risk of bleeding events. This study examined the bleeding and thromboembolic events of rivaroxaban and apixaban. Medical records from 114 patients (rivaroxaban *n* = 64, apixaban *n* = 80) treated for stroke prevention in atrial fibrillation at a tertiary hospital in Malaysia were retrospectively reviewed. Patients with bleeding or stroke/systemic embolism events were identified and the bleeding risk factors were investigated using logistic regression analysis. Stroke or systemic embolism after treatment with Factor Xa (FXa) inhibitor occurred in 12 (8.33%) of the patients, 5 (3.47%) were ischaemic stroke and 7 (4.86%) of them were presented with myocardial infarction. Bleeding occurred in 32 (22.20%) patients, where 7 (4.90%) were presented with major bleeding (rivaroxaban *n* = 3, apixaban *n* = 4), while another 25 (17.40%) experienced clinically relevant non-major bleeding. Furthermore, concomitant antiplatelet used and serum creatinine level were significant predictors of bleeding events (*P* < 0.05). In conclusion, stroke or systemic embolism events were low for both drugs, but this may be an underestimate of the true prevalence due to the small sample size in the present study.

## Introduction

Rivaroxaban and apixaban are Factor Xa (FXa) inhibitor oral anticoagulants that are used to treat and prevent stroke and systemic embolism in patients with non-valvular atrial fibrillation, deep vein thrombosis and pulmonary embolism. As with all anticoagulants, the use of rivaroxaban and apixaban carries an increased risk of bleeding events.

Only two FXa inhibitors (i.e. rivaroxaban and apixaban) are available in Malaysia, but their safety and effectiveness data in the local setting are still limited. Rivaroxaban and apixaban were initially registered in Malaysia in 2011 and 2013, respectively. Based on the preliminary data by the Malaysian National Pharmaceutical Regulatory Agency in 2014, the most reported adverse drug reaction (ADR) was bleeding related events; 59.3% for rivaroxaban, while for apixaban only one report which was gastrointestinal bleeding (1).

Therefore, this study aimed to evaluate the safety and effectiveness of rivaroxaban and apixaban at Universiti Kebangsaan Malaysia Medical Centre, Kuala Lumpur, Malaysia. In addition, risk factors associated with bleeding outcomes in patients receiving rivaroxaban or apixaban were investigated in the present study.

## Methods

### Study Design

This research was a retrospective observational study conducted at Universiti Kebangsaan Malaysia Medical Centre. The list of patients receiving rivaroxaban or apixaban were traced from the computerised pharmacy system. Patients treated for stroke prevention in atrial fibrillation (SPAF) were screened from the system and those who underwent treatment for deep vein thrombosis or pulmonary embolism were excluded. Then, eligible patients were conveniently sampled into the study by reviewing their medical records manually. Data collection was carried out over three and half months from mid-February 2019 until the end of May 2019.

### Data Collection

The data collection form consisted of five parts: i) demographic data such as age, sex, race, weight, height, smoking status and alcohol intake status; ii) comorbidities and concomitant medications; iii) congestive heart failure, hypertension, age ≥75 (doubled), diabetes, stroke (doubled), vascular disease, age 65 to 74 and sex category (female) (CHA2DS2-VASc) score upon initiation and hypertension, abnormal renal/liver function, stroke, bleeding history or predisposition, labile international normalised ratio, elderly, drugs/alcohol concomitantly (HAS-BLED) score; iv) laboratory investigation data which include haemoglobin level, platelet level, renal profile, liver profile and coagulation profile; and v) thromboembolic and bleeding events that were documented after treatment with FXa inhibitor.

### Statistical Analysis

Jamovi v1.6.15 (2) was used to conduct all statistical analyses. Descriptive statistic was used to analyse demographic data. Categorical data such as sex, race, comorbidities, concomitant medications and presence of bleeding or thromboembolic events were presented as frequency and percentage, while continuous variables were presented as mean and standard deviation or median and interquartile range (IQR) depending on normality distribution which was tested using the Kolmogorov-Smirnov equation. Univariate analysis logistic regression was used to screen covariates for significance at *P* < 0.2. Factors and covariates with *P* < 0.2 in the univariate analysis were used in a multivariate logistic regression analysis to identify predictors of bleeding events at the *P* < 0.05 level of significance using a stepwise approach. Factors with probability values of more than 0.20 but high possibility of association as shown by previous studies were also included in the multivariate analysis.

## Results

The baseline demographic and clinical characteristics of the patients are summarised in Table 1. Majority of them were males (56.3%), Chinese (49.3%), with a median [IQR] age of 69.5 years old [63, 74]. Meanwhile, the most common comorbidities recorded were hypertension (90.3%) followed by diabetes mellitus (48.6%). The median congestive heart failure, hypertension, age ≥ 75 years old (doubled), diabetes, stroke/transient ischemic attack/thromboembolism (doubled), vascular disease (prior myocardial infarction, peripheral artery disease, or aortic plaque), age 65 years old–75 years old, sex category (female) (CHA_2_DS_2_-VASc) score for males was 3 and 4 for females, while the overall median CHA_2_DS_2_-VASc score was 3. On the other hand, the median for HASBLED score for all patients was 2. A total of 12 (8.33%) patients presented with thromboembolic events; five patients were presented with recurrent ischaemic stroke and another seven presented with myocardial infarction. There was no significant difference in thromboembolic events between rivaroxaban and apixaban.

A total of 32 bleeding events (14 rivaroxaban and 18 apixaban) were recorded in this study, consisting of seven major bleeding cases, while 25 were clinically relevant non-major (CRNM) bleeding. Three of the major bleeding occurred in rivaroxaban-treated patients while four occurred in apixaban-treated patients. Furthermore, two patients presented with CRNM were categorised as major bleeding because they required blood transfusion due to bleeding and low haemoglobin level. The summary of thromboembolic and bleeding events is summarised in Table 2. The detail characteristic for each patient with thromboembolic events and major bleeding are presented in Supplementary Tables S1 and S2, respectively.

In the univariate analysis, the occurrence of bleeding events was significantly associated with congestive heart failure, chronic kidney disease, concomitant antiplatelet (aspirin or clopidogrel), history of bleeding, baseline haemoglobin level, history of peptic ulcer disease and serum creatinine at the time of bleeding (Table 3). On the other hand, only antiplatelet use and serum creatinine level during the event were significant risk factors for bleeding events in the multiple regression analysis. The use of an antiplatelet was associated with a 4.17 times higher risk of bleeding (adjusted OR: 4.171; 95% CI: 1.18, 14.714; *P* = 0.03). In addition, the serum creatinine level (μmol/L) during the event (indicating possible poor renal function during the event) will increase the risk of bleeding (OR: 1.03; 95% CI: 1.01, 1.05; *P* = 0.003).

## Discussion

Medical records from 144 patients taking rivaroxaban or apixaban for SPAF were reviewed in the present study. When each patient was analysed individually, the majority of those who presented with ischaemic stroke were > 60 years old with more than three comorbidities. Moreover, most of them have CHA_2_DS_2_-VASc score of more than 3, which translates to more than 3.2% increase in strokes each year (3). On the other hand, patients presented with acute myocardial infarction aged between 64 years old and 79 years old with more than two comorbidities, with one patient recording a CHA_2_DS_2_-VASc score of 7.

Bleeding events in the rivaroxaban group were 21.90% and 22.50% in the apixaban group. In the rivaroxaban ROCKET-AF trial, the reported percentage of bleeding was 20.7% (4), whereas the proportion of any bleeding is 25.6% (5) in the apixaban for reduction in stroke and other thromboembolic events in atrial fibrillation (ARISTOTLE) trial; both were comparable with the present study. Besides major bleeding, 17.40% of the patients experienced CRNM bleeding or minor bleeding. In this study, the most frequently documented minor bleeding was haematuria followed by gum bleeding and haematoma. In cases where Xa was administered on patients, no prominent minor bleeding is not expected to occur; nonetheless, the frequency of minor bleeding varies between studies (6–7). It should be noted that documentation of minor bleeding largely depends on patients’ reports. Visually obvious bleeding such as haematuria, haematoma, epistaxis and gum bleeding might be reported more than non-obvious bleeding that is non-clinically apparent such as acute gastritis with bleeding and changes in menstruation frequency or volume.

Antiplatelet use and higher serum creatinine level are associated with increased bleeding risk, thus useful predictors in some studies (8). However, the diagnosis of renal impairment was not reported in this study; instead, it is based on the single value of recorded serum creatinine during patient presentation with the bleeding events. Upon further investigation of these patients, their serum creatinine level exceeded 150 μmol/L. Thus, although the FXa inhibitor is partially cleared by the kidney, there is a possibility of accumulation due to decreased clearance (9). Moreover, antiplatelet usage (aspirin or clopidogrel) was associated with an increased risk of bleeding (10). Other known factors from prior studies that were associated with increased risk of bleeding are increased age, sex (male), diastolic blood pressure ≥ 90 mmHg, history of chronic obstructive pulmonary disease or gastrointestinal bleeding, prior aspirin use and anaemia (10).

Currently, there is a lack of studies on the safety and efficacy of FXa in the Malaysian population, but several clinical studies have been reported on dabigatran. Yap et al. (11) described the clinical experience from a single centre (National Heart Institute of Malaysia) on the reported adverse and bleeding events and reasons for switching anticoagulants. Dabigatran and warfarin usage were compared and it was found that the overall bleeding events were lower in the dabigatran group (12). Furthermore, no thromboembolic events were detected in the cohort within their 340.7 ± 322 day follow up for dabigatran and 410.5 ± 321 day follow up for the warfarin group. However, another study by Yap et al. (13) with a larger sample size found no significant differences in the efficacy and safety of dabigatran and warfarin. In contrast, another study conducted in Malaysia found that the predicted rate for dabigatran-induced major bleeding was low, however, the fatality risk was high (14). As aforementioned, no studies on FXa have been conducted in Malaysia. The first published study on a pair-wise comparison between FXa, dabigatran and warfarin suggested the efficacy of dabigatran and FXa were parallel (15). Nevertheless, apixaban bleeding rates were lower than warfarin, but rivaroxaban bleeding rates were higher than warfarin (15).

There are several limitations in the present study. Firstly, this retrospective study manually screened patients’ medical records; thus, the outcome depended heavily on the physicians’ documentation and patient reporting. Some information such as minor bleeding or if the patient sees a different physician in-between visits might not be documented in the medical records, hence the possibility of underrepresentation in clinical events. Secondly, the small sample size was inadequate for a robust multivariate analysis. Furthermore, the medical records were sampled randomly, which resulted in the possibility of selection bias. Nevertheless, medical records selection was not based on any pre-set criteria, but rather retrieval was of medical records done upon their availability within the study period. In addition, the safety and efficacy between apixaban and rivaroxaban were not compared in this study. A proper comparison between these anticoagulants would require propensity matched-scoring to balance the baseline characteristics and reduce bias due to confounding variables. Unfortunately, this was not possible with a relatively small dataset, and time and resource constraints hampered further data collection. Lastly, the follow up duration varied between patients; thus, some clinical events might not be recorded within a short follow up.

## Conclusion

Thromboembolic events after treatment with rivaroxaban or apixaban were low (8.33%). Bleeding events occurred in ~22% of patients on either rivaroxaban or apixaban and was associated with antiplatelet use and higher serum creatinine during the bleeding event. Most importantly, this study provides valuable data for planning future research on oral anticoagulants, particularly in Malaysia.

## Supplementary Tables

**Table S1:** Characteristic of patients with thromboembolic events

No	Sex, age (years old)	Factor Xa inhibitor	Dosage	Time to event	CHA _2_DS _2_-VASc	HAS-BLED	SCr, μmol/L	Thromboembolic event
1	Male, 66	Apixaban	5mg BD	6 months	4	3	83	MI
2	Male, 65	Apixaban	5mg BD	1 month	3	1	74	MI
3	Male, 79	Apixaban	5mg BD	> 1 year	7	2	119	Ischaemic stroke
4	Female, 62	Apixaban	5mg BD	2 years	4	1	112	Ischaemic stroke
5	Male, 74	Apixaban	5mg BD	3 years	4	1	120	Ischaemic stroke
6	Male, 63	Apixaban	5mg BD	3 months	3	2	130	MI
7	Male, 75	Rivaroxaban	20mg OD	< 1 year	3	2	100	MI
8	Female, 77	Rivaroxaban	20mg OD	< 6 months	5	2	108	MI
9	Male, 60	Rivaroxaban	20mg OD	11 months	3	1	85	MI
10	Female, 71	Rivaroxaban	20mg OD	1 year	4	2	114	MI
11	Male, 60	Rivaroxaban	20mg OD	9 months	3	1	98	Ischaemic stroke
12	Female, 71	Rivaroxaban	20mg OD	4 years	4	2	92	Ischaemic stroke

**Table S2:** Characteristic of patients with major bleeding events

Sex, age (years old)	Concomitant antiplatelet	CHA _2_DS _2_-VASc	HAS-BLED	Drug and dosage	SCr (μmol/L)	Bleed type	Time to bleed (month)
Major bleed							
Male, 52	none	2	1	Apixaban 5mg BD	170	Bleeding from anus and rectum	1.5
Female, 70	aspirin	6	3	Apixaban 5mg BD	188	Intracerebral hemorrhage	11
Male, 72	clopidogrel	3	3	Apixaban 5mg BD	224	Melena	2
Male, 84	none	5	3	Apixaban 5mg BD	154	Melena	11
Male, 63	aspirin plus clopidogrel	3	2	Rivaroxaban 20mg OD	511	Bleeding from anus and rectum	7
Female, 77	aspirin	5	2	Rivaroxaban 20mg OD	194	Melena	10
Male, 73	none	3	2	Rivaroxaban 20mg OD	169	Melena	21
***Minor bleed***							
Male, 66	aspirin and clopidogrel	2	3	Apixaban 5mg BD	76	Bleeding from anus and rectum	5
Female, 61	aspirin	3	2	Apixaban 5mg BD	62	Conjunctival haemorrhage	17
Female, 61	aspirin	3	1	Apixaban 5mg BD	-	Epistaxis	10
Male, 68	aspirin	4	3	Apixaban 5mg BD	138	Gum bleeding	7
Female, 70	none	3	2	Apixaban 5mg BD	73	Gum bleeding	48
Male, 63	aspirin and clopidogrel	3	2	Apixaban 5mg BD	145	Gum bleeding	6
Male, 73	none	3	2	Apixaban 5mg BD	144	Gum bleeding	19
Male, 63	none	4	1	Apixaban 5mg BD	151	Hematoma	13
Female, 67	none	4	2	Apixaban 5mg BD	70	Hematoma	12
Female, 66	aspirin	3	2	Apixaban 5mg BD	-	Hematoma	2
Male, 68	none	4	3	Apixaban 5mg BD	88	Hematuria	7
Female, 62	none	4	1	Apixaban 5mg BD	-	Hematuria	2.2
Male, 67	aspirin	2	2	Apixaban 5mg BD	88	Hematuria	2.1
Male, 76	none	4	2	Apixaban 5mg BD	-	Hemoptysis	19
Male, 60	clopidogrel	2	1	Rivaroxaban 20mg OD	167	Acute gastritis with bleeding	5
Female, 77	none	3	1	Rivaroxaban 20mg OD	65	Gum bleeding	3
Female, 77	aspirin	5	2	Rivaroxaban 20mg OD	86	Hematoma	26
Female, 66	none	4	2	Rivaroxaban 20mg OD	88	Hematoma	6
Female, 70	none	5	3	Rivaroxaban 20mg OD	73	Hematuria	13
Male, 81	aspirin	4	3	Rivaroxaban 20mg OD	468	Hematuria	0.10
Male, 88	none	5	3	Rivaroxaban 20mg OD	94	Hematuria	1.5
Male, 60	none	3	1	Rivaroxaban 20mg OD	178	Hematuria	14
Female, 70	aspirin	6	3	Rivaroxaban 20mg OD	60	Hematuria	1.3
Male, 88	none	5	3	Rivaroxaban 20mg OD	1623	Hematuria	2
Female, 78	aspirin	7	5	Rivaroxaban 20mg OD	158	Hemoptysis	41

## Figures and Tables

**Table 1:** Demographic and clinical characteristics of patients

Characteristics	All *N* = 144 (%)	Rivaroxaban *n* = 64 (%)	Apixaban *n* = 80 (%)
Age (y), median (IQR)	69.5 (63, 74)	70 (63, 75)	69.0 (63, 74)
≤ 54 years old	8 (5.60)		
55 years old–64 years old	34 (23.60)		
65 years old–74 years old	70 (48.60)		
≥ 75 years old	32 (22.20)		
Sex			
Male	81 (56.30)	42 (65.60)	39 (48.80)
Female	63 (43.80)	22 (34.40)	41 (51.20)
Race			
Malay	66 (45.80)	34 (53.10)	32 (40.00)
Chinese	71 (49.30)	27 (42.20)	44 (55.00)
Indian	5 (3.50)	3 (4.70)	2 (2.50)
Others	2 (1.40)	-	2 (2.50)
Medical history			
Diabetes mellitus	70 (48.60)	29 (45.30)	41 (51.30)
Hypertension	130 (90.30)	57 (89.10)	73 (9130)
Congestive cardiac failure	12 (8.30)	2 (3.10)	10 (12.50)
Ischaemic heart disease	59 (41.00)	29 (45.30)	30 (37.50)
Stroke/Transient ischaemic attack	33 (22.90)	15 (23.40)	18 (22.50)
Chronic kidney disease	7 (4.90)	2 (3.10)	5 (6.30)
Peptic ulcer disease	3 (2.10)	-	3 (3.80)
Prior bleeding history	2 (1.40)	-	2 (2.50)
Concomitant medication			
Antiplatelet	43 (29.90)	17 (26.60)	26 (32.50)
ACE-i/ARB	89 (61.80)	38 (59.40)	51 (63.80)
Beta-blockers	108 (75.00)	49 (76.60)	59 (73.80)
Calcium channel blocker	57 (39.60)	28 (43.80)	29 (36.30)
NSAIDs	3 (2.10)	2 (3.10)	1 (1.30)
Statin	116 (80.60)	53 (82.80)	63 (78.80)
CHA_2_DS_2_-VASc score, median (IQR)	3.00 (1.00)	3.00 (2.00)	4.00 (1.00)
HAS-BLED score, median (IQR)	2.00 (1.00)	2.00 (1.00)	2.00 (1.00)

**Table 2:** Thromboembolic and bleeding events after treatment with FXa inhibitor

Outcome	All *N* = 144 (%)	Rivaroxaban *n* = 64 (%)	Apixaban *n* = 80 (%)
Thromboembolic events after treatment with FXa inhibitor			
Yes	12 (8.33)	6 (9.38)	6 (7.50)
No	132 (91.70)	58 (90.62)	74 (92.50)
Type of thromboembolic events			
Ischaemic stroke	5 (3.47)	2 (3.13)	3 (3.75)
Myocardial infarction	7 (4.86)	4 (6.25)	3 (3.75)
Bleeding			
Yes	32 (22.20)	14 (21.90)	18 (22.50)
No	112 (77.80)	50 (78.10)	62 (77.50)
Severity of bleeding			
Major	7 (4.90)	3 (4.70)	4 (5.00)
Clinically relevant non-major bleeding	25 (17.40)	11 (17.20)	14 (17.50)
Major bleeding			
Intracerebral haemorrhage	1 (0.70)	0	1 (1.30)
Melena	4 (2.80)	2 (3.10)	2 (2.50)
CRNM required blood transfusion	2 (1.40)	1 (1.60)	1 (1.30)
Clinically relevant non-major bleeding			
Haematuria	9 (6.30)	6 (9.40)	3 (3.80)
Gum bleeding	5 (3.50)	1 (1.60)	4 (5.00)
Haematoma	5 (3.50)	2 (3.10)	3 (3.80)
Bleeding from anus/rectum	1 (0.70)	0	1 (1.30)
Haemoptysis	2 (1.40)	1 (1.60)	1 (1.30)
Acute gastritis with bleeding	1 (0.70)	1 (1.60)	0
Epistaxis	1 (0.70)	0	1 (1.30)
Conjunctival haemorrhage	1 (0.70)	0	1 (1.30)

**Table 3:** Multiple logistic regression regarding predictors of bleeding outcome in patient on FXa inhibitor

Variables	B	Adjusted OR	95% CI	*χ*^2^ (df)^a^	*P*-value^a^
Comorbidities					
Congestive cardiac failure	0.62	1.86	0.26, 13.14	0.39 (1)	0.53
Chronic kidney disease	−0.27	0.766	0.04, 13.81	0.03 (1)	0.86
Peptic ulcer disease	1.70	5.495	0.22, 137.0	0.31 (1)	0.31
Prior bleeding event	−0.72	0.485	0.00, 383.8	0.05 (1)	0.83
Concomitant medications					
Antiplatelet	1.43	4.17	1.18, 14.7	5.11 (1)	**0.02**
Baseline haemoglobin level	−0.21	0.81	0.59, 1.11	1.87 (1)	0.17
Serum creatinine during the event	0.03	1.03	1.01, 1.05	12.88 (1)	**< 0.001**

Notes: df-degree of freedom;

a Omnibus likelihood ratio test;

* *P*-value < 0.05 denotes significance
